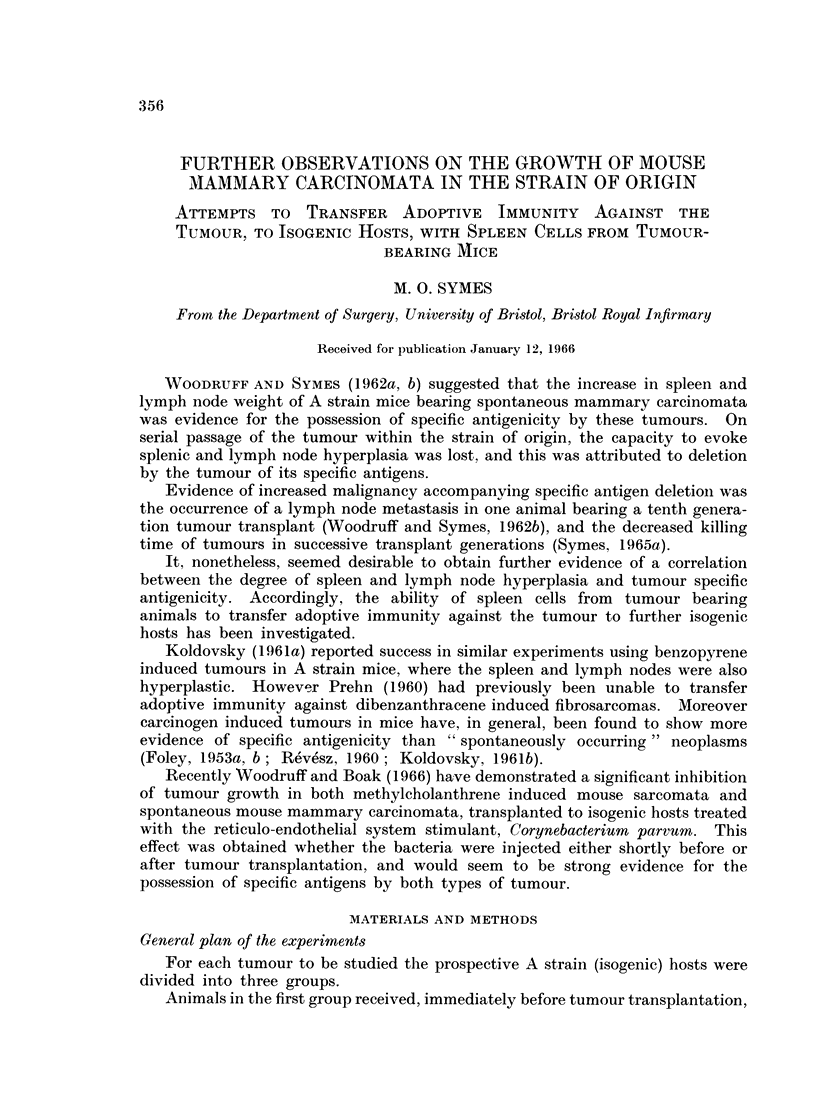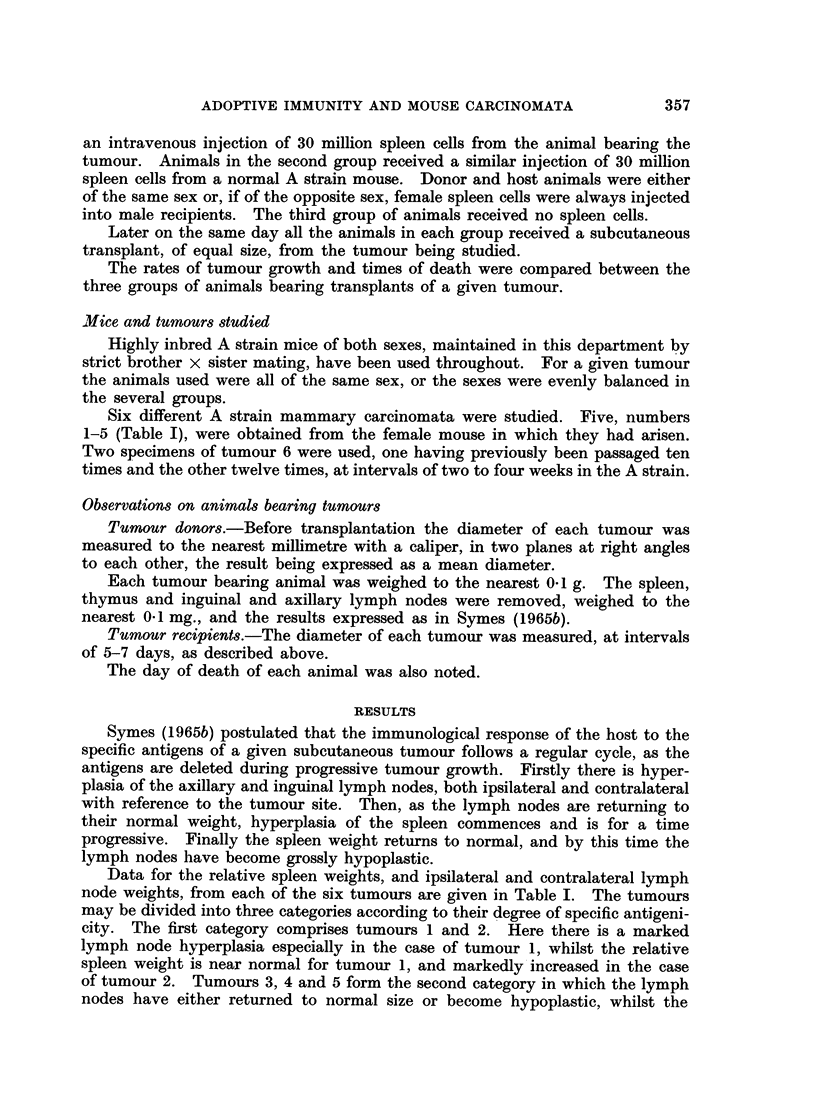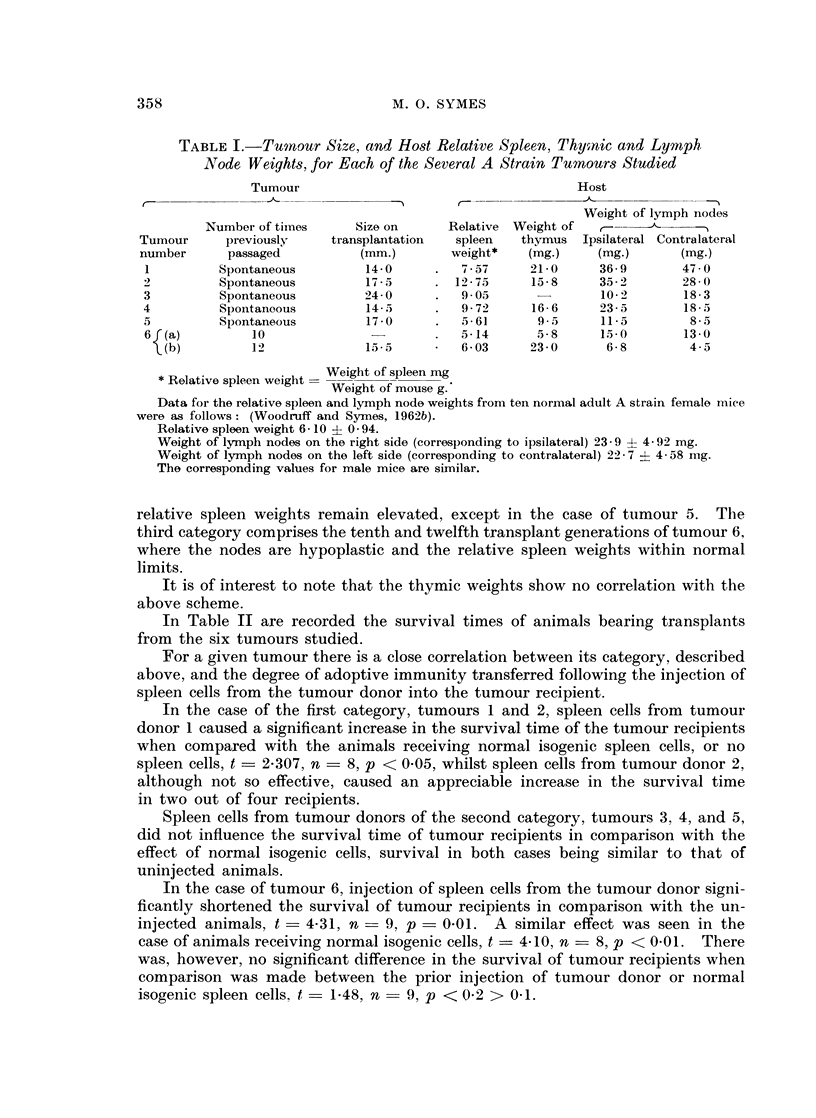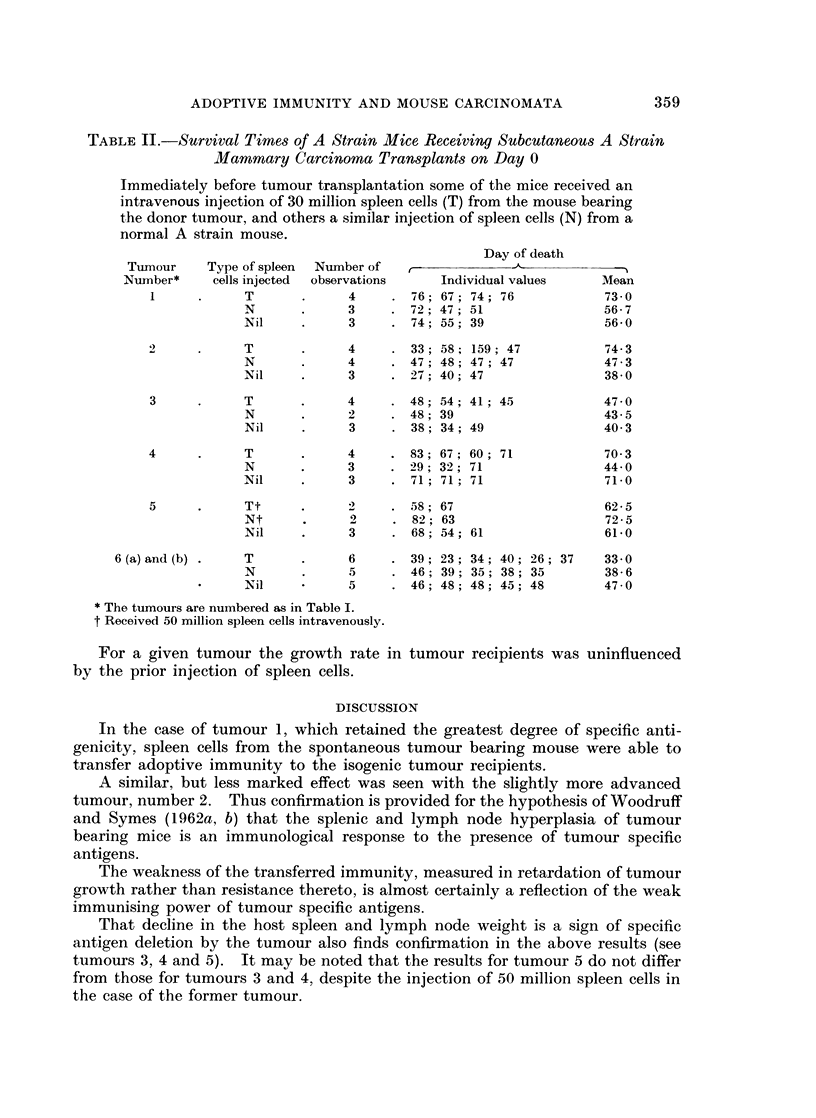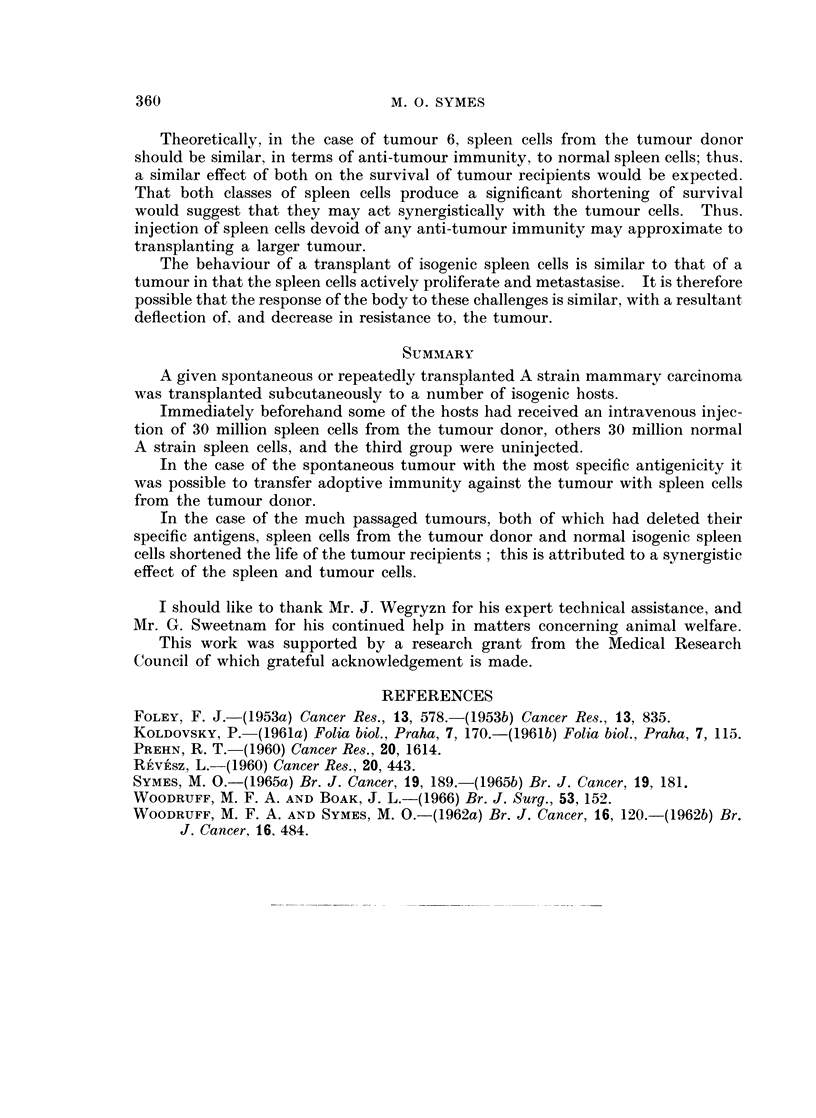# Further observations on the growth of mouse mammary carcinomata in the strain of origin. Attempts to transfer adoptive immunity against the tumour, to isogenic hosts, with spleen cells from tumour-bearing mice.

**DOI:** 10.1038/bjc.1966.43

**Published:** 1966-06

**Authors:** M. O. Symes


### FURTHER OBSERVATIONS ON THE GROWTH OF MOUSE MAMMARY CARCINOMATA IN THE STRAIN OF ORIGIN

### ATTEMPTS TO TRANSFER ADOPTIVE IMMUNITY AGAINST THE TUMOUR, TO ISOGENIC HOSTS, WITH SPLEEN CELLS FROM TUMOUR-BEARING MICE

M. O. SYMES

*From the Department of Surgery, University of Bristol, Bristol Royal Infirmary*

Received for publication January 12, 1966

WOODRUFF AND SYMES (1962*a*, *b*) suggested that the increase in spleen and lymph node weight of A strain mice bearing spontaneous mammary carcinomata was evidence for the possession of specific antigenicity by these tumours. On serial passage of the tumour within the strain of origin, the capacity to evoke splenic and lymph node hyperplasia was lost, and this was attributed to deletion by the tumour of its specific antigens.

Evidence of increased malignancy accompanying specific antigen deletion was the occurrence of a lymph node metastasis in one animal bearing a tenth generation tumour transplant (Woodruff and Symes, 1962*b*), and the decreased killing time of tumours in successive transplant generations (Symes, 1965*a*).

It, nonetheless, seemed desirable to obtain further evidence of a correlation between the degree of spleen and lymph node hyperplasia and tumour specific antigenicity. Accordingly, the ability of spleen cells from tumour bearing animals to transfer adoptive immunity against the tumour to further isogenic hosts has been investigated.

Koldovsky (1961*a*) reported success in similar experiments using benzopyrene induced tumours in A strain mice, where the spleen and lymph nodes were also hyperplastic. However Prehn (1960) had previously been unable to transfer adoptive immunity against dibenzanthracene induced fibrosarcomas. Moreover carcinogen induced tumours in mice have, in general, been found to show more evidence of specific antigenicity than " spontaneously occurring " neoplasms (Foley, 1953*a*, *b* ; Révész, 1960 ; Koldovsky, 1961*b*).

Recently Woodruff and Boak (1966) have demonstrated a significant inhibition of tumour growth in both methylcholanthrene induced mouse sarcomata and spontaneous mouse mammary carcinomata, transplanted to isogenic hosts treated with the reticulo-endothelial system stimulant, *Corynebacterium parvum*. This effect was obtained whether the bacteria were injected either shortly before or after tumour transplantation, and would seem to be strong evidence for the possession of specific antigens by both types of tumour.

### MATERIALS AND METHODS

*General plan of the experiments*

For each tumour to be studied the prospective A strain (isogenic) hosts were divided into three groups.

Animals in the first group received, immediately before tumour transplantation, an intravenous injection of 30 million spleen cells from the animal bearing the tumour. Animals in the second group received a similar injection of 30 million spleen cells from a normal A strain mouse. Donor and host animals were either of the same sex or, if of the opposite sex, female spleen cells were always injected into male recipients. The third group of animals received no spleen cells.

Later on the same day all the animals in each group received a subcutaneous transplant, of equal size, from the tumour being studied.

The rates of tumour growth and times of death were compared between the three groups of animals bearing transplants of a given tumour.

*Mice and tumours studied*

Highly inbred A strain mice of both sexes, maintained in this department by strict brother × sister mating, have been used throughout. For a given tumour the animals used were all of the same sex, or the sexes were evenly balanced in the several groups.

Six different A strain mammary carcinomata were studied. Five, numbers 1–5 (Table I), were obtained from the female mouse in which they had arisen. Two specimens of tumour 6 were used, one having previously been passaged ten times and the other twelve times, at intervals of two to four weeks in the A strain.

*Observations on animals bearing tumours*

*Tumour donors.*—Before transplantation the diameter of each tumour was measured to the nearest millimetre with a caliper, in two planes at right angles to each other, the result being expressed as a mean diameter.

Each tumour bearing animal was weighed to the nearest 0·1 g. The spleen, thymus and inguinal and axillary lymph nodes were removed, weighed to the nearest 0·1 mg., and the results expressed as in Symes (1965*b*).

*Tumour recipients.*—The diameter of each tumour was measured, at intervals of 5–7 days, as described above.

The day of death of each animal was also noted.

### RESULTS

Symes (1965*b*) postulated that the immunological response of the host to the specific antigens of a given subcutaneous tumour follows a regular cycle, as the antigens are deleted during progressive tumour growth. Firstly there is hyperplasia of the axillary and inguinal lymph nodes, both ipsilateral and contralateral with reference to the tumour site. Then, as the lymph nodes are returning to their normal weight, hyperplasia of the spleen commences and is for a time progressive. Finally the spleen weight returns to normal, and by this time the lymph nodes have become grossly hypoplastic.

Data for the relative spleen weights, and ipsilateral and contralateral lymph node weights, from each of the six tumours are given in Table I. The tumours may be divided into three categories according to their degree of specific antigenicity. The first category comprises tumours 1 and 2. Here there is a marked lymph node hyperplasia especially in the case of tumour 1, whilst the relative spleen weight is near normal for tumour 1, and markedly increased in the case of tumour 2. Tumours 3, 4 and 5 form the second category in which the lymph nodes have either returned to normal size or become hypoplastic, whilst the

TABLE I.—*Tumour Size, and Host Relative Spleen, Thymic and Lymph Node Weights, for Each of the Several A Strain Tumours Studied*

| Tumour | | | Host | | | |
|---|---|---|---|---|---|---|
| | | | | | Weight of lymph nodes | |
| Tumour number | Number of times previously passaged | Size on transplantation (mm.) | Relative spleen weight* | Weight of thymus (mg.) | Ipsilateral (mg.) | Contralateral (mg.) |
| 1 | Spontaneous | 14·0 | 7·57 | 21·0 | 36·9 | 47·0 |
| 2 | Spontaneous | 17·5 | 12·75 | 15·8 | 35·2 | 28·0 |
| 3 | Spontaneous | 24·0 | 9·05 | — | 10·2 | 18·3 |
| 4 | Spontaneous | 14·5 | 9·72 | 16·6 | 23·5 | 18·5 |
| 5 | Spontaneous | 17·0 | 5·61 | 9·5 | 11·5 | 8·5 |
| 6 (a) | 10 | — | 5·14 | 5·8 | 15·0 | 13·0 |
| 6 (b) | 12 | 15·5 | 6·03 | 23·0 | 6·8 | 4·5 |

$$\text{* Relative spleen weight} = \frac{\text{Weight of spleen mg}}{\text{Weight of mouse g.}}$$

Data for the relative spleen and lymph node weights from ten normal adult A strain female mice were as follows: (Woodruff and Symes, 1962*b*).
Relative spleen weight 6·10 ± 0·94.
Weight of lymph nodes on the right side (corresponding to ipsilateral) 23·9 ± 4·92 mg.
Weight of lymph nodes on the left side (corresponding to contralateral) 22·7 ± 4·58 mg.
The corresponding values for male mice are similar.

relative spleen weights remain elevated, except in the case of tumour 5. The third category comprises the tenth and twelfth transplant generations of tumour 6, where the nodes are hypoplastic and the relative spleen weights within normal limits.

It is of interest to note that the thymic weights show no correlation with the above scheme.

In Table II are recorded the survival times of animals bearing transplants from the six tumours studied.

For a given tumour there is a close correlation between its category, described above, and the degree of adoptive immunity transferred following the injection of spleen cells from the tumour donor into the tumour recipient.

In the case of the first category, tumours 1 and 2, spleen cells from tumour donor 1 caused a significant increase in the survival time of the tumour recipients when compared with the animals receiving normal isogenic spleen cells, or no spleen cells, $t = 2{\cdot}307$, $n = 8$, $p < 0{\cdot}05$, whilst spleen cells from tumour donor 2, although not so effective, caused an appreciable increase in the survival time in two out of four recipients.

Spleen cells from tumour donors of the second category, tumours 3, 4, and 5, did not influence the survival time of tumour recipients in comparison with the effect of normal isogenic cells, survival in both cases being similar to that of uninjected animals.

In the case of tumour 6, injection of spleen cells from the tumour donor significantly shortened the survival of tumour recipients in comparison with the uninjected animals, $t = 4{\cdot}31$, $n = 9$, $p = 0{\cdot}01$. A similar effect was seen in the case of animals receiving normal isogenic cells, $t = 4{\cdot}10$, $n = 8$, $p < 0{\cdot}01$. There was, however, no significant difference in the survival of tumour recipients when comparison was made between the prior injection of tumour donor or normal isogenic spleen cells, $t = 1{\cdot}48$, $n = 9$, $p < 0{\cdot}2 > 0{\cdot}1$.

TABLE II.—*Survival Times of A Strain Mice Receiving Subcutaneous A Strain Mammary Carcinoma Transplants on Day 0*

Immediately before tumour transplantation some of the mice received an intravenous injection of 30 million spleen cells (T) from the mouse bearing the donor tumour, and others a similar injection of spleen cells (N) from a normal A strain mouse.

| Tumour Number* | Type of spleen cells injected | Number of observations | Day of death: Individual values | Day of death: Mean |
|---|---|---|---|---|
| 1 | T | 4 | 76; 67; 74; 76 | 73·0 |
| | N | 3 | 72; 47; 51 | 56·7 |
| | Nil | 3 | 74; 55; 39 | 56·0 |
| 2 | T | 4 | 33; 58; 159; 47 | 74·3 |
| | N | 4 | 47; 48; 47; 47 | 47·3 |
| | Nil | 3 | 27; 40; 47 | 38·0 |
| 3 | T | 4 | 48; 54; 41; 45 | 47·0 |
| | N | 2 | 48; 39 | 43·5 |
| | Nil | 3 | 38; 34; 49 | 40·3 |
| 4 | T | 4 | 83; 67; 60; 71 | 70·3 |
| | N | 3 | 29; 32; 71 | 44·0 |
| | Nil | 3 | 71; 71; 71 | 71·0 |
| 5 | T† | 2 | 58; 67 | 62·5 |
| | N† | 2 | 82; 63 | 72·5 |
| | Nil | 3 | 68; 54; 61 | 61·0 |
| 6 (a) and (b) | T | 6 | 39; 23; 34; 40; 26; 37 | 33·0 |
| | N | 5 | 46; 39; 35; 38; 35 | 38·6 |
| | Nil | 5 | 46; 48; 48; 45; 48 | 47·0 |

* The tumours are numbered as in Table I.
† Received 50 million spleen cells intravenously.

For a given tumour the growth rate in tumour recipients was uninfluenced by the prior injection of spleen cells.

### DISCUSSION

In the case of tumour 1, which retained the greatest degree of specific antigenicity, spleen cells from the spontaneous tumour bearing mouse were able to transfer adoptive immunity to the isogenic tumour recipients.

A similar, but less marked effect was seen with the slightly more advanced tumour, number 2. Thus confirmation is provided for the hypothesis of Woodruff and Symes (1962*a*, *b*) that the splenic and lymph node hyperplasia of tumour bearing mice is an immunological response to the presence of tumour specific antigens.

The weakness of the transferred immunity, measured in retardation of tumour growth rather than resistance thereto, is almost certainly a reflection of the weak immunising power of tumour specific antigens.

That decline in the host spleen and lymph node weight is a sign of specific antigen deletion by the tumour also finds confirmation in the above results (see tumours 3, 4 and 5). It may be noted that the results for tumour 5 do not differ from those for tumours 3 and 4, despite the injection of 50 million spleen cells in the case of the former tumour.

Theoretically, in the case of tumour 6, spleen cells from the tumour donor should be similar, in terms of anti-tumour immunity, to normal spleen cells; thus, a similar effect of both on the survival of tumour recipients would be expected. That both classes of spleen cells produce a significant shortening of survival would suggest that they may act synergistically with the tumour cells. Thus, injection of spleen cells devoid of any anti-tumour immunity may approximate to transplanting a larger tumour.

The behaviour of a transplant of isogenic spleen cells is similar to that of a tumour in that the spleen cells actively proliferate and metastasise. It is therefore possible that the response of the body to these challenges is similar, with a resultant deflection of, and decrease in resistance to, the tumour.

### Summary

A given spontaneous or repeatedly transplanted A strain mammary carcinoma was transplanted subcutaneously to a number of isogenic hosts.

Immediately beforehand some of the hosts had received an intravenous injection of 30 million spleen cells from the tumour donor, others 30 million normal A strain spleen cells, and the third group were uninjected.

In the case of the spontaneous tumour with the most specific antigenicity it was possible to transfer adoptive immunity against the tumour with spleen cells from the tumour donor.

In the case of the much passaged tumours, both of which had deleted their specific antigens, spleen cells from the tumour donor and normal isogenic spleen cells shortened the life of the tumour recipients ; this is attributed to a synergistic effect of the spleen and tumour cells.

I should like to thank Mr. J. Wegryzn for his expert technical assistance, and Mr. G. Sweetnam for his continued help in matters concerning animal welfare.

This work was supported by a research grant from the Medical Research Council of which grateful acknowledgement is made.

### REFERENCES

Foley, F. J.—(1953*a*) *Cancer Res.*, **13**, 578.—(1953*b*) *Cancer Res.*, **13**, 835.

Koldovsky, P.—(1961*a*) *Folia biol., Praha*, **7**, 170.—(1961*b*) *Folia biol., Praha*, **7**, 115.

Prehn, R. T.—(1960) *Cancer Res.*, **20**, 1614.

Révész, L.—(1960) *Cancer Res.*, **20**, 443.

Symes, M. O.—(1965*a*) *Br. J. Cancer*, **19**, 189.—(1965*b*) *Br. J. Cancer*, **19**, 181.

Woodruff, M. F. A. and Boak, J. L.—(1966) *Br. J. Surg.*, **53**, 152.

Woodruff, M. F. A. and Symes, M. O.—(1962*a*) *Br. J. Cancer*, **16**, 120.—(1962*b*) *Br. J. Cancer*, **16**, 484.